# Availability of informal social support and the impact on health services utilization among women in community corrections who engage in substance use and risky sexual behavior: New York City, 2009–2012

**DOI:** 10.1186/s40352-022-00170-0

**Published:** 2022-02-16

**Authors:** Karli R. Hochstatter, Melissa N. Slavin, Louisa Gilbert, Dawn Goddard-Eckrich, Nabila El-Bassel

**Affiliations:** School of Social Work, Columbia University, 1255 Amsterdam Ave, New York, NY 10027 USA

**Keywords:** Justice-involved women, Community corrections, Substance use, Social support, Health services research

## Abstract

**Background:**

Women under community supervision in the U.S. experience high rates of substance use and HIV/STDs and face multiple barriers to healthcare services. Informal social support, provided by family, friends, and other peers, is important for reducing drug and sexual risk behaviors and improving utilization of healthcare services. The availability of informal social support and the impact on receipt of healthcare services among the growing and highly vulnerable population of sexually-active and drug- and justice-involved women has not been documented. Among this population, this study aims to: 1) describe characteristics of informal social support, including the prevalence of different types, size of networks, and frequency of receiving support; and 2) longitudinally examine the impact of informal social support on receipt of healthcare services, including drug or alcohol counseling/treatment, HIV or STD counseling/education, birth control counseling/education, reproductive healthcare, and individual counseling over a 12-month period.

**Results:**

The sample included 306 women in community supervision programs in New York, New York, USA, with a recent history of substance use and risky sexual behavior. At baseline, 96.1% of women reported having at least one friend or family member with whom they could discuss personal or emotional problems, 92.5% had support for tangible aid or service, 83.0% had support for sexual risk reduction, and 80.0% had support for substance use risk reduction. Women with support for substance use risk reduction were more likely than women without this type of support to receive all health services analyzed in this study. Having support for sexual risk reduction was also positively associated will receipt of all services, except reproductive healthcare. Having support for personal or emotional problems was only associated with receiving drug or alcohol counseling or treatment, while having support for tangible aid or service did not impact receipt of any health services.

**Conclusions:**

Engagement of sexually-active and drug- and justice-involved women in health services should address the availability and strengthening of informal social support, particularly ensuring individuals’ informal networks allow for discussions on the harms of risky sexual and drug use behaviors.

**Trial registration:**

ClinicalTrials.govNCT01784809. Registered 6 February 2013 - Retrospectively registered.

## Background

### Overrepresentation of women in correctional systems

Women represent a growing proportion of the correctional population in the United States.

Approximately 82% of women under supervision of the U.S. correctional system are in community corrections programs, which include probation, parole, drug treatment courts and other alternative-to-incarceration programs. Although there are approximately 3.5 times as many men under community supervision than women, women make up a growing portion of arrests (Prison Policy Initiative, 2019). As of 2017, women accounted for 27% of all arrests, up from 16% in 1980, while men accounted for 73% of all arrests, down from 84% in 1980 (Prison Policy Initiative, 2019). As a result, the number of women under community supervision in the U.S. has nearly doubled from 520,000 in 1990 to nearly 1 million at year-end 2019. Women now account for 25% of the probation population and 13% of the parole population (Bureau of Justice Statistics, 2020; The Pew Charitable Trusts, 2018). The increase in arrests of women is primarily due to expansive law enforcement efforts and stricter drug sentencing laws (The Sentencing Project, 2020). From 2013 to 2017, drug arrests increased 25% among women, but only 6% among men (Federal Bureau of Investigation, 2018). Furthermore, racialized drug laws and policing practices have resulted in a vast overrepresentation of Black women in community supervision programs (Bailey et al., 2017; Kunins, 2020).

### Substance use disorders and HIV among women in correctional systems

Research also demonstrates that incarcerated women are more likely than men to use drugs associated with a higher risk of overdose and addiction, such as crack/cocaine and heroin, and use them more frequently than men (Langan & Pelissier, 2001). It is estimated that approximately 45% of women under community supervision have a substance use disorder (The Pew Charitable Trusts, 2018), which is a well-established risk factor for human immunodeficiency virus (HIV) and other sexually-transmitted diseases (STDs) (Elkbuli et al., 2019). HIV prevalence rates among women who use drugs mandated to community corrections in New York City are estimated to range from 13% to 17% (Belenko et al., 2004; El-Bassel et al., 2017), which are comparable to rates found among women in sub-Saharan Africa, the region of the world most severely affected by the HIV epidemic (Ramjee & Daniels, 2013). The prevalence of other STDs among substance-using women in community corrections is estimated at 26% (El-Bassel et al., 2017). These women often have low levels of HIV knowledge (Belenko et al., 2004; Browne-Marshall, 2012; Gordon et al., 2013), and due to exposure to a spectrum of overlapping risk contexts (Belenko et al., 2004; Hammett et al., 2002; Spaulding et al., 2002), criminal justice-involved women often engage in unprotected sex with multiple partners; trade sex for money, drugs, or a place to stay (Epperson et al., 2011; Khan et al., 2008); and experience high rates of intimate partner violence (IPV) and low levels of condom use (El-Bassel et al., 2010, 2017). Women in community corrections who use drugs and are sexually active face additional risks, including unintended and high-risk pregnancies and other reproductive health issues (Clarke et al., 2006; Crandall et al., 2003; Fogel, 1993). To improve the health of this high-risk population, it is critical they receive equitable access to health services that address these co-occurring and synergistic health concerns, including drug and alcohol counseling/treatment, HIV/STD counseling/treatment, birth control counseling/education, and reproductive health care.

### Barriers to health services among women in correctional systems

Women in community corrections who have substance use issues face multiple barriers to accessing health services, including lack of health insurance, high rates of relapse, and economic hardship (Gordon et al., 2013; Lorvick et al., 2015). Women under community supervision, particularly women who have recently released from incarceration, face several competing priorities that draw attention away from their health, including housing, employment, obtaining food, and parenting (Ramaswamy et al., 2015; Youmans et al., 2013). These barriers result in limited use of health services among criminal justice-involved women, including services for substance use, HIV/STDs, and women’s health services such as well-women exams and reproductive health care, while increasing the use of hospital emergency departments for chronic and minor health care (Abbott et al., 2017; Bandara et al., 2015; Smith et al., 2019). Research also suggests that women with substance use disorder are more likely than men to face multiple barriers to accessing health care services, including substance abuse treatment (Brady & Randall, 1999; Brady & Ashley, 2005; Hecksher & Hesse, 2009), exacerbating the barriers to care that women under community supervision face. Understanding factors that facilitate use of health services for women in community corrections programs who use drugs may assist in better supporting their wellbeing and decreasing their risk of overdose, HIV/STD transmission, unintended pregnancy, pregnancy and other reproductive health complications, and recidivism.

### Social support as a facilitator to health services

Social support is an important facilitator for improving utilization of health services and preventing SUD relapse and re-incarceration, especially for women (Abbott et al., 2017; Benda, 2005; Chen, 2010; Liau et al., 2004; Parsons & Warner-Robbins, 2002). Informal social support is support provided from family, friends, neighbors, or other peers in one’s social network, and may be classified based on its function and structure. Although there are vast differences in how researchers identify and measure informal social support, there is a broad consensus that four main constructs encompass the different types of support that can be given: informational support (e.g., exchange of advice, suggestions, and information), emotional support (e.g., expressions of empathy, love, trust, and caring), instrumental support (e.g., tangible aid and service), and appraisal support (e.g., being listened to) (Campbell et al., 2011; Langford et al., 1997). These constructs may also be problem-specific, such as support for substance use or mental health issues (Groh et al., 2007, 2008; Muñoz-Laboy et al., 2014). Studies focused on the general population of women have demonstrated that informal social support is associated with lower drug and alcohol use (Boyd & Mieczkowski, 1990; Maisto et al., 1999), substance use treatment entry and retention (Dobkin et al., 2002), positive treatment outcomes (Comfort et al., 2003; Joe et al., 2002; Zywiak et al., 2002), and HIV testing and treatment initiation and adherence (Edwards, 2006; Waddell and Messeri, 2006; Warren-Jeanpiere et al., 2014). Very few studies have examined the association between social support and health services utilization among women in community corrections. Informal social support and social networks have been shown, however, to be more important for utilization of healthcare services among justice-involved women than men (Nowotny, 2016). Furthermore, a study on women in probation programs found that greater informal social support was related to fewer lifetime and 12-month sexual partners (Engstrom et al., 2017).

### Current study

Although it has been well-documented that informal social support improves engagement in health services, there are currently no studies documenting availability of informal social support and the impact of support on receipt of healthcare services among the growing and highly vulnerable population of sexually-active women under community supervision who use drugs. Understanding how social support impacts health services utilization among this population will inform the development of interventions that leverage social support networks to link justice-involved women to healthcare in the community and reduce the harmful consequences of risky drug use and sexual behaviors. Data from this study will also inform substance use treatment practices and healthcare delivery among this population by understanding the importance of involving informal network members in treatment plans to improve linkage and retention in services. This study examines informal social support for: 1) personal or emotional problems; 2) tangible aid or service; 3) sexual risk reduction; and 4) substance use risk reduction, among sexually-active and drug- and justice-involved women. Examining these social support constructs separately will inform intervention strategies that target networks with varying structures and functions. The objectives of this study are to: 1) describe characteristics of informal social support, including the prevalence of each type, number of family members and friends to provide each type, and frequency of receiving each type of support among 306 women under community supervision in New York City who participated in an HIV intervention study; and 2) longitudinally examine the association between informal social support and utilization of health services, including drug or alcohol counseling/treatment, HIV or STD counseling/education, birth control counseling/education, reproductive health care, and individual counseling. Because social networks and support often fluctuate among people with substance use disorders and criminal justice involvement (Lander et al., 2013; Pettus-Davis et al., 2017), this paper uses data collected at four time points: 0, 3, 6, and 12 months. The proposed mechanisms by which social support influences health are typically demonstrated by two theoretical frameworks: the buffering model (Aneshensel & Stone, 1982), which states that social support networks protect against the negative impacts of stressors, and the direct effects model (Wheaton, 1985), which holds that social support can also be beneficial in the absence of stressors. Based on these theoretical models and prior research documenting the positive association between informal social support and utilization of health services, we hypothesize that each type of social support (i.e., personal/emotional, tangible/service, sexual risk reduction, and substance use risk reduction) will be positively associated with having received each health service (i.e., drug or alcohol counseling/treatment, HIV or STD counseling/education, birth control counseling/education, reproductive health care, and individual counseling) among sexually-active women under community supervision who use drugs.

## Methods

### Study design and population

This study was completely embedded in Women on the Road to Health (WORTH), a randomized controlled trial (RCT) that assessed the effectiveness of a group-based HIV and IPV prevention intervention among sexually-active women with substance use issues mandated to community corrections. The RCT was conducted in New York City between November 2009 and January 2012 and collected data at baseline and 3-, 6-, and 12-month follow-ups. To be eligible to participate, women had to report: 1) being aged 18 years or older; 2) being mandated to community corrections (i.e., probation, parole, community court, drug treatment court, or an alternative-to-incarceration program) in the past 90 days; 3) using illicit drugs, binge drinking, or attending a substance abuse treatment program in the past 90 days; 4) engaging in unprotected vaginal or anal intercourse within the past 90 days; and 5) having at least one other HIV risk factor. Potential participants were considered ineligible if they were unable to complete the informed consent process due to a psychiatric or cognitive impairment, unable to speak English, or if they were actively trying to become pregnant. Women with pregnancy intentions were excluded due to the intervention’s emphasis on condom use. Women who did not have an address where they could receive mail, lived more than 90 min from New York City, or planned to move more than 90 min outside of New York City were also excluded. All participants provided written consent to participate in the study. Data were collected at each timepoint using audio computer-assisted self-interview (ACASI) software at a centrally located community research office. Data used in this secondary analysis was collected from all women who participated in the RCT. Additional details of the RCT, including the intervention’s effectiveness, have been published elsewhere (El-Bassel et al., 2014; Gilbert et al., 2016).

### Measurements

*Sociodemographic variables.* Women self-reported sociodemographic characteristics including age, race, ethnicity, marital status, educational attainment, employment status, monthly income, homelessness, mental health diagnoses, the types of community corrections settings where they had enrolled in the past 90 days, the number of times they had been arrested or incarcerated in jail or prison in the past 90 days, and substances used in the past 90 days, including alcohol, marijuana, and other illicit substances.

#### Social support measures

To understand general and problem-specific social support for sexually-active women under community supervision who use drugs, the survey included 11 social support items, which were then collapsed into four types of informal social support (see Table 1) that describe support for: personal or emotional problems; tangible aid or service; sexual risk reduction; and substance use risk reduction. This scale was constructed for use in the original RCT to measure the extensiveness of participants’ social support networks and determine to what extent they utilized informal social support. At each timepoint, women were asked how many friends or family members they had to provide each of the 11 items (continuous variable) in the past 90 days. Because we were unable to determine whether support for each item was provided by the same social network member or different, social support for each type was dichotomized into two groups for regression analyses (0 = no friends or family members to provide that type of support; 1 = at least one friend or family member to provide that type of support). Women were also asked how frequently they received each of the 11 support items in the past 90 days, on a scale from 0 to 6 (0 = never, 1 = once, 2 = twice, 3 = 3 to 5 times, 4 = 6 to 10 times, 5 = 11–20 times, 6 = 20+ times).

**Table 1:** The four types of informal social support analyzed and the corresponding survey questions

Social Support Types	Survey Items: *“Had a friend or family member to …*”
**Personal or emotional problems**	1) talk to when they feel upset or angry; 2) ask advice about personal problems they may be having; 3) talk to about relationship problems they may be having with their partner;
**Tangible aid or service**	4) ask to borrow money when they need it; 5) ask to stay in their place for a while; 6) ask for help with a task that takes at least four hours of their time (e.g. helping with a move, taking care of their kids, cleaning their apartment);
**Sexual risk reduction**	7) talk to about ways they can reduce their risk for HIV or STDs; 8) talk to about the need to use condoms to protect themselves against HIV or STDS;
**Substance use risk reduction**	9) talk to about ways they can reduce sharing syringes, cookers, cotton, or rinse water to avoid transmitting HIV or Hepatitis C; 10) talk to about ways they can reduce or stop using drugs; 11) talk to about ways they can prevent overdose.

#### Healthcare service utilization outcomes

Past 90-day utilization of five different types of health services were assessed at each timepoint (0, 3, 6, and 12 months): 1) drug or alcohol counseling or treatment; 2) HIV or STD counseling or education; 3) birth control counseling or education; 4) reproductive health care; and 5) individual counseling. Individuals were asked whether they received each type of service in the past 90 days and response options included 1 = yes or 0 = no.

### Statistical analysis

Descriptive statistics were used to describe the availability of social support, the size of support networks, and the frequency of receiving social support at baseline. Mixed effects logistic regression models were used to determine the effect of social support on receipt of each type of health service. To accommodate and adjust for the correlation of observations within persons (i.e., repeated measures), we included a random intercept for Study ID, which is a widely used analytical approach for longitudinal data (Gunasekara et al., 2014; Madssen et al., 2021). A separate model was used for each of the four social support types. Each model included robust standard errors to account for potential heteroskedasticity, and adjusted for timepoint, age, randomization arm (control or intervention group), and whether they were under community supervision at the time of the follow-up survey. These control variables were selected because women’s health and therefore healthcare needs likely depend on age, whether they received the intervention that aimed to prevent health risks, and whether they were enrolled in a probation or parole program, which may assist clients with linkage to health services. We also tested the association between each type of health service and other factors that may be barriers to service utilization, including employment status, health insurance status, income, and whether they have children. Because we did not detect any statistically significant associations, and because our sample size is not large enough to include all covariates in the mixed effect models, we did not include these variables in the final models. Additionally, because social networks and support often fluctuate among people with substance use issues and criminal justice involvement (Lander et al., 2013; Pettus-Davis et al., 2017), we treated our primary independent variable, social support, as a time-variant indicator (i.e., social support was reassessed at each timepoint). Statistical significance was assessed using the associated 95% confidence interval and 2-tailed α = 0.05 for each estimate. All statistical analyses were performed using Stata, version 16 (StataCorp LLC).

## Results

### Sociodemographic characteristics

A total of 306 women enrolled in Project WORTH and were randomized (103 received computerized WORTH, 101 received traditional WORTH, and 102 received the wellness intervention). Table 2 describes the sociodemographic characteristics at baseline for the study population. The mean age was 41.5 years. The sample was 73% Black and 18% Hispanic or Latino. Fifty-eight percent had a high school diploma or GED and 66% were single at the time of enrollment. A small proportion of the sample was employed (8%), made >$850 per month (11%), and homeless in the past 90 days (9%). Ninety-one percent were ever incarcerated in jail or prison. Mental health illnesses were highly prevalent, with 61% diagnosed with depression, 49% with anxiety, and 32% with bipolar disorder. At baseline, 23% were mandated to community courts, 35% were on probation, 13% were on parole, 15% were in a drug or mental health court, and 8% were in an alternative-to-incarceration program. Among the 306 women enrolled, 267 (87%) were followed-up at month 3, 277 (91%) were followed-up at month 6, and 278 (91%) were follow-up at month 12. At 3, 6, and 12 months, 164 (61%), 185 (67%), and 209 (75%) women had released from community supervision, respectively.

**Table 2:** Socio-demographic characteristics of project WORTH participants at baseline: New York City, 2009–2012; *N* = 306

Baseline Characteristic	Total (***n*** = 306)
WORTH Study Arm, n (%)	
Wellness	102 (33.3)
Traditional WORTH	101 (33.0)
Computerized WORTH	103 (33.7)
Age (years), mean (SD)	41.5 (10.5)
Black or African American, n (%)	222 (72.6)
Hispanic or Latino, n (%)	55 (18.0)
American Indian, n (%)	6 (2.0)
High School or GED, n (%)	176 (57.5)
Marital Status, n (%)	
Single	202 (66.0)
Married	49 (16.0)
Divorced/separated/widowed	55 (18.0)
Has children, n (%)	232 (75.5)
Has medical insurance, n (%)	278 (90.9)
Employed, n (%)	25 (8.2)
Monthly Income, n (%)	
< $400	176 (57.5)
$400 to $850	95 (31.0)
> $850	35 (11.4)
Had a regular place to sleep (past 90 days), n (%)	267 (87.3)
Incarcerated in the past 90 days, n (%)	72 (23.5)
Community court (past 90 days), n (%)	70 (22.9)
On probation (past 90 days), n (%)	107 (35.0)
On parole (past 90 days), n (%)	40 (13.1)
Drug or mental health court (past 90 days), n (%)	47 (15.4)
Alternative-to-incarceration program (past 90 days), n (%)	23 (7.5)
Mental Health Diagnoses (lifetime), n (%)	
Depression	188 (61.4)
Anxiety	149 (48.7)
Bipolar Disorder	99 (32.4)
Schizophrenia	29 (9.5)
Other mental health illness	56 (18.3)
Used any illicit substance (past 90 days), n (%)	147 (48.0)
Type of substance used (past 90 days), n (%)	
4 or more alcoholic drinks in period of 6 h	93 (30.4)
Marijuana	117 (38.2)
Heroin	54 (17.7)
Cocaine	122 (39.9)
Crack	95 (31.0)
Methamphetamine	2 (0.65)
“Uppers”	8 (2.6)
“Downers”	33 (10.8)
Non-prescribed opiates	29 (9.5)
Ecstasy	20 (6.5)
Other types of drugs	7 (2.3)
HIV-positive^a^, n (%)	43 (14.1)

^a^Two individuals refused to share the result of their most recent HIV test

### Social support

The availability of different types of social support, size of each support network, and frequency of receiving support at baseline for the 11 social support item assessed in this study are displayed in Table 3. At baseline, 96.1% (293/305) of women reported having at least one friend or family member with whom they could discuss personal or emotional problems, 92.5% (282/305) had social support for tangible aid or service, 83.0% (253/305) had social support for sexual risk reduction, and 80.0% (244/305) had social support for substance use risk reduction. Network size and frequency of receiving support was greatest for social support of personal or emotional problems. At baseline, 219/305 (71.8%) women reported having social support across all four social support types, while 9/305 (3.0%) women reported not having any type of social support, 8/305 (2.6%) women had one type of support, 22/305 (7.2%) women had two types of support, and 48/305 (15.7%) women had three types of support. To examine overlap among specific support types, we present the number of women who had support for each combination of support types, at baseline, in Table 4. These social support characteristics remained relatively consistent over time and did not significantly differ between women who remained under community supervision and those who had been released over the course of the study.

**Table 3:** Baseline social support characteristics among women who use drugs and are under community supervision (past 90 days): New York City, 2009–2012; N=305^a^

Type of Social Support	n (%) of women who have ≥ 1 friend or family member to provide this type of social support	Median (IQR) number of friends/family members available for each type of social support	Median (IQR) describing how often this type of support was received in the past 90 days, on a scale from 0 to 6^**b**^
**Personal or emotional problems**			
1. Someone to talk to when they feel upset or angry	286 (93.8)	3 (2–5)	4 (3–6)
2. Someone to ask advice about personal problems	282 (92.5)	3 (2–5)	3 (2–5)
3. Someone to talk to about relationship problems	268 (87.9)	2 (1–3)	2 (1–4)
**Tangible aid and service**			
4. Someone to ask to borrow money when they need it	260 (85.3)	2 (1–3)	2 (1–3)
5. Someone to ask to stay in their place for a while	228 (74.8)	2 (0–3)	0 (0–2)
6. Someone to ask for help with a task that takes at least four hours of their time	229 (75.1)	2 (1–4)	1 (0–2)
**Sexual risk reduction**			
7. Someone to talk to about ways they can reduce their risk for HIV or STDs	235 (77.1)	2 (1–4)	1 (0–3)
8. Someone to talk to about the need to use condoms to protect themselves against HIV or STDS	223 (73.1)	2 (0–4)	1 (0–3)
**Substance use risk reduction**			
9. Someone to talk to about ways they can reduce sharing syringes, cookers, cotton, or rinse water to avoid transmitting HIV or Hepatitis C	166 (54.4)	1 (0–3)	1 (0–3)
10. Someone to talk to about ways they can reduce or stop using drugs	234 (76.7)	2 (1–5)	2 (0–4)
11. Someone to talk to talk about ways they can prevent overdose	181 (59.3)	1 (0–4)	1 (0–3)

^a^Social support information was missing for one individual at baseline

^b^Scale: 0 = never, 1 = once, 2 = twice, 3 = 3 to 5 times, 4 = 6 to 10 times, 5 = 11–20 times, 6 = 20+ times

**Table 4:** The number (percent) of women who had social support for each combination of support types, at baseline (past 90 days): New York City, 2009–2012; *N* = 305

	Personal or emotional problems	Tangible aid or service	Sexual risk reduction	Substance use risk reduction
Personal or emotional problems		279 (91.5%)	252 (82.6%)	243 (79.7%)
Tangible aid or service			244 (80.0%)	237 (77.7%)
Sexual risk reduction				225 (73.8%)
Substance use risk reduction				

### Receipt of healthcare services

#### Substance use treatment or counseling services

At months 0, 3, 6, and 12, 61.3%, 46.0%, 41.9%, and 35.9% of women reported receiving substance use treatment or counseling services in the past 90 days, respectively. Women who reported having social support for personal or emotional problems were 3.22 times more likely to report receiving substance use treatment or counseling services (OR: 3.22; 95% CI: 1.15–9.04; *P* = 0.026) compared to those with no social support for personal or emotional problems. Likewise, women who reported having social support for sexual risk reduction were 1.81 times more likely (OR: 1.81; 95% CI: 1.08–3.04; *P* = 0.025), and women who reporting having social support for substance use risk reduction were 2.02 times more likely (OR: 2.02, 95% CI: 1.24–3.31, *P* = 0.005), to report receiving substance use treatment or counseling services, compared to women without each respective social support. There was no significant difference in receipt of any health services between those who reporting having social support for tangible aid or service and those who did not (Table 5).

**Table 5:** The impact of social support on receipt of healthcare services: New York City, 2009–2012

	Adjusted^**a**^ Odds Ratio (95% Confidence Interval) of Receiving each Health Service				
Type of Social Support	Drug or alcohol counseling/ treatment	HIV or STD counseling or education	Birth control counseling or education	Reproductive health care	Individual counseling
Personal or emotional problems	3.22 (1.15–9.04) *	1.34 (0.62–2.90)	1.04 (0.47–2.31)	1.10 (0.52–2.34)	1.53 (0.68–3.46)
Tangible aid or service	1.15 (0.58–2.30)	1.08 (0.65–1.80)	1.32 (0.74–2.34)	1.07 (0.61–1.89)	1.34 (0.71–2.53)
Sexual risk reduction	1.81 (1.08–3.04) *	1.61 (1.02–2.55) *	1.83 (1.17–2.84) *	1.31 (0.84–2.05)	1.85 (1.19–2.88) *
Substance use risk reduction	2.02 (1.24–3.31) *	1.92 (1.29–2.86) *	1.77 (1.17–2.66) *	1.62 (1.08–2.43) *	2.01 (1.33–3.03) *

*Statistically significant at *P* < 0.05

^a^Longitudinal mixed-effects logistic regression adjusted for timepoint, age, randomization arm, and whether they were under community supervision in the past 90 days

#### HIV or STD counseling or education

At months 0, 3, 6, and 12, 55.7%, 54.0%, 46.6%, and 43.1% of women reported receiving HIV or STD counseling or education in the past 90 days, respectively. Women who reported having social support for sexual risk reduction were 1.61 times more likely (OR: 1.61; 95% CI: 1.02–2.55; *P* = 0.041), and women who reporting having social support for substance use risk reduction were 1.92 times more likely (OR: 1.92, 95% CI: 1.29–2.86, *P* = 0.001), to report receiving HIV or STD counseling or education, compared to women without each respective social support.

#### Birth control counseling or education

At months 0, 3, 6, and 12, 32.5%, 28.7%, 31.1%, and 25.0% of women reported receiving birth control counseling or education in the past 90 days, respectively. Women who reported having social support for sexual risk reduction were 1.83 times more likely (OR: 1.83; 95% CI: 1.17–2.84; *P* = 0.008), and women who reporting having social support for substance use risk reduction were 1.77 times more likely (OR: 1.77, 95% CI: 1.17–2.66, *P* = 0.007), to report receiving birth control counseling or education, compared to women without each respective social support.

#### Reproductive health care

At months 0, 3, 6, and 12, 35.4%, 39.6%, 37.6%, and 30.4% of women reported receiving reproductive health care in the past 90 days, respectively. Women who reported having social support for substance use risk reduction were 1.62 times more likely to report receiving reproductive health care (OR: 1.62; 95% CI: 1.08–2.43; *P* = 0.021) compared to those with no social support for substance use risk reduction.

#### Individual counseling

At months 0, 3, 6, and 12, 55.4%, 47.9%, 44.0%, and 40.6% of women reported receiving individual counseling in the past 90 days, respectively. Women who reported having social support for sexual risk reduction were 1.85 times more likely (OR: 1.85; 95% CI: 1.19–2.88; *P* = 0.006), and women who reporting having social support for substance use risk reduction were 2.01 times more likely (OR: 2.01, 95% CI: 1.33–3.03, *P* = 0.001), to report receiving individual counseling, compared to women without each respective social support.

## Discussion

### Principal findings

The goal of this paper was to fill a gap in the literature by characterizing informal social support among sexually-active and drug- and justice-involved women in community corrections, and examine the impact of social support on receipt of various health services. The types of informal social support examined in this study were highly prevalent among the study population. Although most women reported having at least one friend or family member to provide social support, those without social support were significantly less likely to receive a variety of health services. Women with informal social support for substance use risk reduction were significantly more likely than women without this type of social support to receive all health services analyzed in this study, including drug or alcohol counseling or treatment, HIV or STD counseling or education, birth control counseling or education, and individual counseling. Having social support for sexual risk reduction was also positively associated with receipt of all health services, except reproductive health care. Although the strongest effect observed, having social support for personal or emotional problems was only associated with receiving drug or alcohol counseling or treatment. We found that having friends and family to provide tangible aid or service does not significantly impact receipt of any of the health services assessed in this study. These positive associations between informal social support and health services utilization support our hypotheses and align with previous studies that have described the important role that family, friends, and other peers play in improving engagement in healthcare among the general population of women (Boyd & Mieczkowski, 1990; Comfort et al., 2003; Dobkin et al., 2002; Edwards, 2006; Joe et al., 2002; Maisto et al., 1999; Waddell and Messeri, 2006; Warren-Jeanpiere et al., 2014; Zywiak et al., 2002). These findings suggest that informal networks that allow for discussions on the harms of risky sexual and drug use behaviors play an important role in the uptake of health care services that address these harms.

### Implications

Interventions should be developed and implemented that focus on strengthening and expanding informal social relationships among justice-involved women at risk of the harmful consequences from risky substance use and sexual behaviors. As outlined in a literature review, interventions that target social support can be implemented in a variety of ways to achieve positive outcomes (Hogan et al., 2002). Some examples of these formats include group interventions with family members or relationship partners, which can teach individuals new coping skills to build their relationships and work on issues together. There are also group interventions that provide support through peers (self-help groups), which allow participants to provide and receive support, develop friendships, and rebuild lasting social networks after a crisis (e.g., Alcoholics Anonymous; Narcotics Anonymous). Additionally, there are individual interventions that target social skills such as those that focus on changing the behavior of the patient, while recruiting a friend or family member to provide emotional support and positive reinforcement. Individual interventions can also provide support through peers (e.g., peer support interventions), where the patient is paired with someone from the community who has had similar experiences and can serve as a confidant and help the individual through a particular experience or issue. Individual interventions by trained professionals (e.g., social workers, nurses) can also provide emotional support, informational support, and/or instrumental support. Lastly, there are individual interventions that focus on teaching social skills to unassertive individuals through use of rehearsal, modeling, instruction, and behavioral feedback. A combination of these interventions described (e.g., combined group and individual therapy; combined provision of support and social skills training) can also be implemented. Interventions are particularly successful if they are matched to the specific population and reciprocal support is emphasized (e.g., both giving and receiving support) (Hogan et al., 2002). Additionally, individuals may be particularly likely to seek out and benefit from social support for issues which are considered more stigmatizing (e.g., substance use disorders, HIV) (Davison et al., 2000).

In this current study, women with social support in one area (e.g., substance use risk reduction) were significantly more likely than women without this type of social support to receive health services in other areas beyond drug or alcohol counseling or treatment (e.g., HIV or STD counseling or education, birth control counseling or education, and individual counseling.) Thus, interventions that focus on obtaining social support in even one area can have multiple benefits for women in other areas. This is particularly important, as there are overlapping risk contexts (Belenko et al., 2004; Hammett et al., 2002; Spaulding et al., 2002) among women involved in criminal justice systems, with one risk behavior (e.g., substance use) increasing the likelihood for others (e.g., HIV and STIs; intimate partner violence; unintended and high-risk pregnancies (Clarke et al., 2006; Crandall et al., 2003; El-Bassel et al., 2010, 2017; Elkbuli et al., 2019). Research has established that professional treatment in one of these areas can lead to improvement in other areas (e.g., substance use treatment improving intimate partner violence outcomes) (Murphy & Ting, 2010; Parcesepe et al., 2020), but these current findings suggest that even informal methods of support in one area have the potential to impact other areas of health.

### Strengths

This study enrolled women in community corrections who recently used substances, engaged in unprotected sexual intercourse, had at least 1 other HIV risk factor, and did not have pregnancy intentions. These inclusion criteria provided a cohort whom could all benefit from the health services analyzed in this study. This is also an incredibly vulnerable population that is often hard-to-reach, yet this study enrolled a sizeable cohort (*n* = 306) and had impressively high retention rates (91% at month 12). Although self-report measures were used to assess the study variables, these measures were administered through audio, computer-assisted technology that presumably lessened threats of respondent biases. Additionally, the analyses tested multiple measures of social support and included multiple covariates.

### Limitations

This study is not without limitations. One limitation is that this is a secondary data analysis of a RCT that enrolled women into an HIV and IPV prevention intervention study. Consequently, the study was not designed, and therefore powered, to detect the associations examined in this paper. Furthermore, this sample may be biased towards women with stronger interests in engaging in risk reduction and, as such, they may have higher social support associated with these motivations. Another limitation is that we did not use a validated tool to measure informal social support. The items included in our study were developed for the purposes of the original RCT and did not allow us to measure receipt of informational support nor appraisal support, two of the four main informal social support constructs typically measured by researchers (Campbell et al., 2011; Langford et al., 1997). Our study did, however, examine emotional support and instrumental support. Additionally, receipt of each health service assessed was based on a single survey question. Thus, we could not determine, for example, how informal social support impacts receipt of different types of substance use treatments (e.g., group-based counseling, individual therapy, medication-assisted treatments, etc.) or length of service engagement.

This study also assumed that the need for each of these health services remained constant over the 12-month study period. However, it is possible that some women changed their behavior, removed persons from their informal network whom they discussed that behavior with, and no longer needed services related to that behavior. For example, women may have stopped using substances, removed persons who use drugs from their network (i.e. people they would talk to about substance use risk reduction), and were no longer in need of treatment or counseling. However, when we controlled for whether women binge drank, used marijuana, or used other illicit substances in the past 90 days, the association between social support for substance use risk reduction and receipt of drug or alcohol counseling or treatment services remained positive and statistically significant. Similarly, when we controlled for whether women had sex without a condom in the past 90 days, the association between social support for sexual risk reduction and receipt of HIV or STD counseling or education remained the same. Lastly, when examining the outcomes of receipt of birth control counseling or education and receipt of reproductive health care, the results remained consistent when controlling for women who decided they wanted to get pregnant during the study period. These additional analyses strengthen the validity of this study’s results.

## Conclusions

Women under community supervision experience high rates of substance use and HIV/STDs and face multiple barriers to healthcare services. Women who lack informal social support from family members, friends, or other peers are particularly unlikely to receive and benefit from substance use, HIV/STD, and reproductive health services. Effective and gender-specific strategies that aim to connect and sustain retention of sexually-active and drug- and justice-involved women in health care services should address the availability and strengthening of informal social support, particularly ensuring individuals’ informal networks allow for discussions on the harms of risky sexual and drug use behaviors.

## Data Availability

The datasets generated and/or analyzed during the current study are not publicly available but are available from the corresponding author on reasonable request.

## Abbreviations

STD: Sexually-transmitted disease
HIV: Human immunodeficiency virus
IPV: Intimate partner violence
RCT: Randomized controlled trial
